# Neural Circuit Mechanisms of General Anaesthesia Induction: Current Advances and Future Directions

**DOI:** 10.2174/011570159X387168250912070856

**Published:** 2025-09-24

**Authors:** Yanfang Yin, Yaxin Teng, Wenying Chi, Xinyuan Zhang, Haozhe Qiao, Xiaoyong Zhao, Meiyan Sun

**Affiliations:** 1 School of Anaesthesiology, Shandong Second Medical University, Weifang, Shandong Province, China;; 2 Department of Anesthesiology, Jinan Central Hospital, Jinan, China;; 3 Laboratory of Anaesthesia and Critical Care Medicine in Colleges and Universities of Shandong Province, School of Anaesthesiology, Shandong Second Medical University, Weifang, Shandong Province, China;; 4 Department of Anaesthesiology, Shandong Cancer Hospital and Institute, Shandong First Medical University and Shandong Academy of Medical Sciences, Jinan, Shandong Province, China

**Keywords:** Consciousness, general anaesthesia, neural circuit, peripheral nervous system, spinal cord, loss of consciousness

## Abstract

General anaesthesia is traditionally divided into three distinct stages: induction, maintenance, and recovery. However, much of the existing literature has primarily focused on elucidating the mechanisms involved in the recovery phase, yielding several notable advancements. It is essential to recognize, however, that the induction and recovery phases represent two distinct processes. Studies in the induction phase have mainly centred on the impact of inhalational and intravenous anaesthetics on neural circuits, particularly those in the cortical and subcortical nuclei, as well as their specific effects on various neurotransmitters. Yet, the precise neural circuit mechanisms underlying anaesthetic induction still require further exploration. General anaesthetics influence neural circuitry by targeting neurons in particular nuclei, with their effects varying according to the distinct properties of individual anaesthetic agents. During the induction of anaesthesia, both the cortex and subcortical nuclei are significantly involved, with the inhibition of the subthalamic nucleus considered a core mechanism underlying this process. Notably, the periventricular thalamus, as part of the thalamus structure, holds particular importance in regulating the loss of consciousness. Additionally, the spinal cord and peripheral nervous system may play a potentially important role during the induction phase of general anaesthesia. Gaining a deeper understanding of the mechanisms underlying anaesthetic induction could reveal potential neuroanatomical targets that elucidate the alterations in consciousness during this phase of general anaesthesia. Such insights are invaluable in the quest for more effective, precise, and controllable anaesthetic practices, thereby enhancing the selection and combination of anaesthetic agents.

## 1 INTRODUCTION

General anesthesia is a drug-induced, reversible state of coma, characterized by unconsciousness, amnesia, pain relief, loss of sensation, and suppression of spinal motor reflexes, while maintaining stability in the autonomic, cardiovascular, respiratory, and thermoregulatory systems [1-3]. This state ensures that the patient loses consciousness, remains pain-free during surgery, and maintains the necessary stability to ensure surgical safety. The process of general anaesthesia includes induction, maintenance, and recovery phases. Research indicates that anaesthesia induction and recovery are two separate processes [4] with compelling evidence highlighting the critical role of ascending arousal pathways during recovery [5]. Furthermore, there is a significant need for additional investigation into the neural pathways implicated in the induction phase.

The mechanisms by which general anaesthetics exert their effects may involve altering cell membrane permeability, binding to specific protein targets, or interacting with membrane-mediated targets. These actions disrupt neural activity and compromise the integrity and functionality of neural circuits that facilitate signal transmission and conscious perception/subjective experience [6]. The neural circuit theory offers a crucial perspective, emphasising that general anaesthetics induce and maintain unconsciousness by influencing neuronal networks and signal transmission within the nervous system. Specifically, these drugs may enhance the action of inhibitory neurotransmitters or inhibit the release of excitatory neurotransmitters, affecting neural activity in key brain regions such as the cortex and hypothalamus. This involves not only the responses of individual neurons but also the overall regulation and balance of neural circuits.

Research has indicated that different anaesthetics may have distinct underlying mechanisms related to molecular targets and neural circuits during the induction process [6]. Although many studies are based on animal models, the selection of literature on neural circuit mechanisms in this review is limited to those studies that provide feasible data for investigating the induction of anaesthesia in humans [7]. This review examines the neural circuitry mechanisms involved in the induction of general anesthesia. By analysing the effects of anaesthetic agents on specific neural pathways, it aims to deepen our understanding of the dynamic processes involved in anaesthetic induction and reveals the significant role of the peripheral nervous system in sensory processing and information transmission, providing new directions for future research. This perspective offers valuable insights into the pursuit of more efficient, precise, and controllable anaesthetic practices.

This review article utilized the PubMed database for literature retrieval. Structured Boolean search strategies were employed, combining key terms related to consciousness, general anesthesia, induction, mechanism, neural circuit, subcortical nuclei (*e.g*., thalamus), major anesthetic agent classes (*e.g*., volatile anesthetics, intravenous anesthetics), and other relevant concepts. The search was explicitly focused on identifying research articles and reviews published in English within the past two decades. Strict inclusion criteria mandated that studies specifically investigate the neural circuit mechanisms underlying the induction phase of general anesthesia. Consequently, studies primarily focused on the emergence/recovery phase, non-neural mechanisms, or significantly deviating themes, as well as non-English publications, were systematically excluded. The analysis specifically examined the distinct pharmacological profiles of different anesthetic classes. It elucidated the central role of key subcortical nuclei and their associated neural circuits in mediating the loss of consciousness during induction.

## 2 EFFECTS OF GENERAL ANAESTHETICS ON NEURAL CIRCUITS

General anaesthetics are primarily classified into volatile anaesthetics and intravenous anaesthetics. The mechanisms of these drugs during the induction phase predominantly focus on inducing loss of consciousness. Inhibition of frontal-parietal connections may indicate a broader disruption of cortical signal connectivity, leading to loss of consciousness. The mechanisms of general anesthesia are currently being studied in relation to cortical and subcortical nuclei [8]. Clinical studies have shown that, during general anaesthesia, there is a preferential suppression of frontal-parietal feedback connections [9]. Anaesthetics with different molecular targets have been observed to induce disruptions in frontal-parietal connections and block information transmission in humans. This may represent a common pathway and a direct cause of anaesthetic-induced loss of consciousness [10-14]. Both volatile and intravenous anaesthetics reduce functional connectivity between the prefrontal cortex, posterior parietal cortex, and cingulate cortex, and they concentrate functional connectivity in brain regions with stronger structural connections [15-17].

Various theories have been proposed to explain consciousness and the mechanisms of general anaesthesia. It has been confirmed that, during general anaesthesia, anaesthetic drugs affect the activity of neural circuits by modulating key structures in the brain, such as the cortex, thalamus, and brainstem, leading to the loss of consciousness. These drugs act on specific neural circuits, such as the cortico-thalamic and cortico-brainstem circuits, altering the excitability of neurons and synaptic transmission, and influencing particular receptors, including GABAergic receptors and NMDA receptors [18]. However, the neural circuit mechanisms between cortical and subcortical nuclei during the induction phase remain ambiguous. Although many studies compare general anaesthesia with natural sleep, as both involve reversible unconsciousness, they are not entirely analogous. The actions and effects on neural circuits vary among different anesthetics. Recent advances in neuroscience have enabled research into the brain nuclei and neural circuits involved [19, 20] primarily involving crucial brain areas such as the brainstem and hypothalamus.

### 2.1 Volatile Anaesthetics

Since the successful introduction of ether for surgical anaesthesia in the 18th century, there has been a remarkable evolution in volatile anaesthetics, transitioning from ether and chloroform to commonly used agents like isoflurane, enflurane, and sevoflurane. Substantial advancements have been achieved in understanding the neural circuits involved in the induction processes of isoflurane and sevoflurane.

#### 2.1.1 Isoflurane

During anaesthesia with the volatile anaesthetic isoflurane, several brainstem nuclei play a crucial role in regulating anaesthetic induction. For example, the locus coeruleus (LC) is involved in cortical arousal. Studies have shown that exposure to isoflurane inhibits activity in the LC, thereby preventing arousal induced by hypothalamic orexin/hypocretin [21]. In recent years, increasing attention has been given to the hypnotic effects of LC and noradrenaline. The induction of isoflurane anaesthesia is delayed by the activation of noradrenergic neurons in the LC [22]. Additionally, recent research suggests that the chemical activation of LC-tyrosine-hydroxylase (TH) neurons promotes cortical arousal, potentially mediated by the activation of the paraventricular thalamus (PVT) [23]. However, it remains unclear whether the LC contributes to anaesthesia induction through other brain nuclei. The pontine reticular formation (PnO) in the brain stem is another critical site for isoflurane anaesthesia. Hypnosis during anesthesia is regulated by GABAergic neurotransmission in the PnO; suppression of GABA neuron activity shortens the induction time, whereas heightened activity of acetylcholine neurons exerts the converse influence, prolonging the duration of anesthesia [21, 24]. During anaesthesia induced by isoflurane, there is a reduction in calcium signalling within dopamine neurons located in the ventral periaqueductal gray (vPAG). Furthermore, the administration of a GABA-A receptor agonist into the vPAG accelerates the induction of anaesthesia and prolongs the recovery period from anaesthesia [25]. Furthermore, anaesthetic potentiation has been experimentally demonstrated to be mediated through the pharmacologically induced activation of GABAergic neurons localized within both the ventral tegmental area (VTA) and the rostral midbrain tegmental complex (RMTg) [26]. VTA GABA neurons facilitate induction by projecting to the lateral hypothalamus (LH) during isoflurane anaesthesia [27]. However, one study revealed that bilateral VTA lesions did not significantly affect the induction of isoflurane [28]. Serotonergic neurons in the dorsal raphe nucleus (DRN) exhibit decreased calcium activity during isoflurane induction and recover after inhalation cessation, suggesting that DRN serotonergic neurons primarily influence recovery rather than induction [29].

During continuous isoflurane induction, cholinergic neuron activity in the basal forebrain (BF) gradually decreases, mediating loss of consciousness, including loss of righting reflex (LORR) [30]. In contrast, growth hormone-releasing peptide β-expressing GABA neurons (GABA^SOM^) in the BF promote hypnosis during isoflurane anaesthesia [31]. The induction time of isoflurane anaesthesia is shortened, and the recovery time from anaesthesia is prolonged, following the activation of astrocytes in the BF [32]. Furthermore, Gao and colleagues [33] found that GABAergic neurons in the nucleus accumbens (NAC) of the BF are activated during isoflurane exposure, paradoxically extending the anesthetic induction phase. Additionally, adenosine (AD) in the BF regulates the hypnotic process of anaesthesia induction [21, 34]. The parabrachial nucleus (PB) does not significantly influence anaesthesia induction [35]. During propofol and isoflurane anesthesia, neuronal activity in the PB is suppressed; activating the PB does not alter the induction time but shortens the recovery time. Crucially, isoflurane-mediated unconsciousness appears to be fundamentally dependent on the engagement of the ventrolateral preoptic nucleus (VLPO) within hypothalamic circuitry, with anaesthetic potentiation primarily occurring through activation of VLPO sleep-promoting neurons [36-38]. Researchers have confirmed that both the VLPO and the median preoptic nucleus (MnPO) [34, 39] contain neurons necessary for promoting non-rapid eye movement (NREM) sleep. However, the specific neuronal activity of the MnPO during anaesthesia remains unclear, further suggesting that anaesthesia cannot be simply equated with natural sleep.

**Fig. (1):**
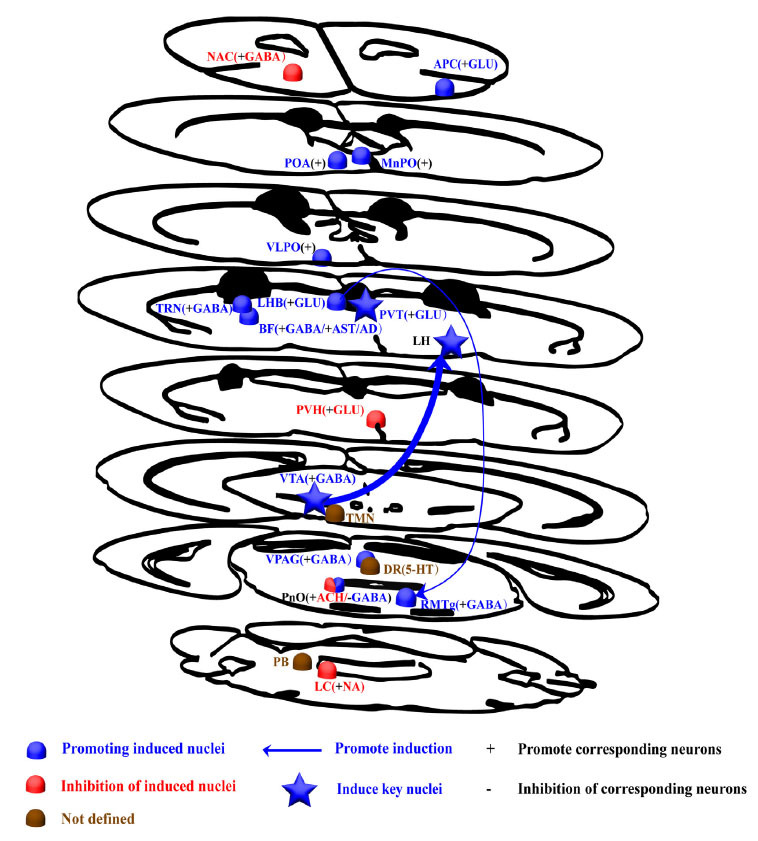
Neuronal connections between cortical and subcortical nuclei during isoflurane-induced general anaesthesia. Within the PnO, GABA neurons facilitate the induction process under isoflurane anaesthesia, while ACH neurons inhibit it.

Lesions in the tuberomammillary nucleus (TMN) increase sensitivity to isoflurane anaesthesia but do not affect sensitivity to other anaesthetics such as propofol, pentobarbital, or ketamine [40]. During isoflurane anesthesia, the induction phase is prolonged, and the depth of anesthesia is enhanced by the activation of glutamatergic neurons in the hypothalamic paraventricular nucleus (PVH) [41]. Furthermore, the corticotropin-releasing factor neurons in this region have been shown to play a role in the awakening from isoflurane anaesthesia [42], although their specific impact on the induction process remains unclear. Chemical stimulation of glutamatergic neurons in the lateral habenula (LHb) has been shown to potentiate anaesthetic induction, whilst optogenetic stimulation of RMTg projections through the LHb-RMTg pathway significantly reduces induction latency [43]. Notably, the LHb is one of the targets of AAN [44]. In the thalamus, the thalamic reticular nucleus (TRN) stands as the sole structure comprised of GABAergic neurons, forming a distinct neuronal nucleus. Engaging GABAergic neurons in the TRN, especially in its anterior part, facilitates anaesthesia induction and delays awakening [45]. During isoflurane anaesthesia, the loss of consciousness is facilitated and the recovery of consciousness is prolonged by inhibiting glutamatergic neurons or their pathways in the PVH. In contrast, sustained activation of neurons in the PVT markedly accelerates the induction of anaesthesia [46]. These interactions between brain regions and neurons determine the induction process of isoflurane anaesthesia. Furthermore, it was shown that isoflurane may also influence synaptic transmission from the periphery to the spinal cord [47]. Altered neuronal activity in demyelinated mice [48] during isoflurane anaesthesia provides a new direction for research into the mechanisms of general anaesthesia (Fig. **1**).

#### 2.1.2 Sevoflurane

Sevoflurane, a volatile anaesthetic, acts on the VTA in the brainstem, which is crucial for the induction of anaesthesia. Selective optogenetic stimulation of dopaminergic (DA) neurons in the VTA is sufficient to induce arousal from unconsciousness or anaesthesia [49]. Real-time fiber photometry data show that calcium signaling in VTA DA neurons significantly decreases during the LORR induced by sevoflurane anaesthesia and significantly increases during recovery of righting reflex (RORR). Activation of VTA-DA neurons, the VTA-DA-NAc pathway [50], and the VTA-prefrontal cortex (PrL) pathway extends induction time [51, 52]. The PrL, as a critical area of the medial prefrontal cortex (PFC), receives dopaminergic projections from the VTA. Integrated chemogenetic and optogenetic approaches have demonstrated that activation of the VTA DA-PrL pathway significantly prolongs the latency of sevoflurane anaesthetic induction [53]. These findings paradoxically suggest that the hypnotic effects of volatile anaesthetics may be related to inhibition of VTA neurons. However, damage to VTA dopaminergic neurons does not affect isoflurane anaesthesia processes [28]. The NAc, located in the ventral striatum, receives projections from VTA dopaminergic neurons, indicating that the presence of these neurons is not essential for anaesthesia induction. Unconsciousness induced by sevoflurane is promoted by GABAergic neurons in the RMTg *via* inhibitory projections to multiple arousal-promoting nuclei [54]. Chemically inhibiting glutamatergic neurons in the pedunculopontine tegmental nucleus (PPTg) accelerates the induction of sevoflurane anesthesia [55]. When D_1_ receptor neurons in the NAc (NAc D_1_R) of the BF are activated chemically or optogenetically, the induction time of sevoflurane anaesthesia in mice is prolonged, while the emergence time is shortened [56]. During sevoflurane anaesthesia, NAc D_1_R neurons and their connections in the ventral pallidum (VP) are inhibited, with a reversible decrease in extracellular GABA levels in the VP during the induction phase [57]. Conversely, the shortening of sevoflurane anaesthesia induction time occurs not only when dopamine D_2_ receptor neurons located in the NAc (NAc D_2_R) are activated [58] but also when the NAc D2R-VP pathway is optogenetically activated [33]. Under sevoflurane anaesthesia, the NAc and adjacent nuclei are in an inhibited state.

**Fig. (2):**
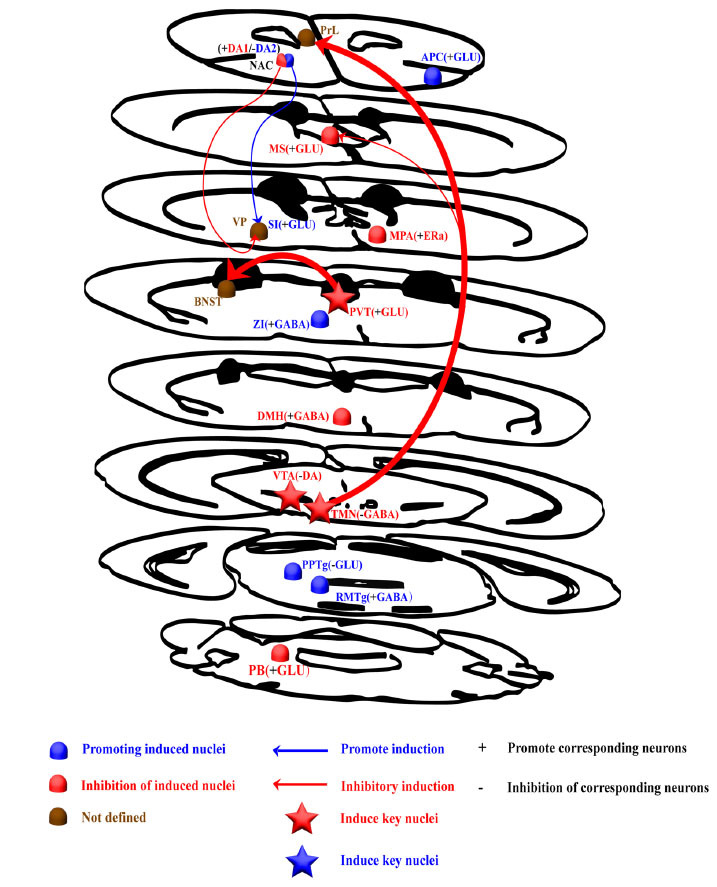
Neuronal connections between cortical and subcortical nuclei during sevoflurane-induced anaesthesia. Within the NAC, DA2 neurons facilitate induction and enhance VP during sevoflurane anaesthesia, while DA1 neurons have the opposite effect.

Recent studies utilizing calcium fiber photometry and other techniques demonstrate that chemical stimulation of glutamatergic neurons in the substantia innominata (SI) facilitates anaesthesia induction [59]. Studies examining sex differences in mice have revealed that estrogen receptor α in the medial preoptic area (MPA) eliminates sex differences in sevoflurane anaesthesia by extending the induction time [60]. Chemogenetic studies in adult mice have shown that activation of glutamatergic neurons in the medial septum (MS) extends the induction time under sevoflurane anesthesia and accelerates the recovery from anesthesia [61]. Activating GABAergic neurons in the TMN diminishes the anaesthetic effects of sevoflurane [62]. Another study found that activation of GABAergic neurons in the dorsomedial hypothalamus (DMH) also resulted in prolonged induction time under sevoflurane anaesthesia [63]. Significant differences in the reduction of arousal levels due to lesions in the medial parabrachial nucleus (MPB) and the lateral parabrachial nucleus (LPB) within the PB [64, 65] indicate that the MPB is primarily involved in promoting wakefulness. Electrophysiological and behavioral analyses indicate that sevoflurane induces hypnosis by inhibiting MPB neurons through GABA-A receptors located postsynaptically and by modulating background potassium channels. Effective inhibition of MPB neurons is crucial for sevoflurane-induced hypnosis [66] which may be one of the main brain targets for sevoflurane anaesthesia. It has been demonstrated that glutamatergic neurons in the PBN prolong induction time only when activated under sevoflurane anaesthesia [67, 68]. This suggests that these neurons may represent a critical target involved in the induction process of sevoflurane anaesthesia. During sevoflurane anesthesia, activating glutamatergic neurons in the PB through chemogenetics results in a longer induction time [67]. Recent studies report that the regulation of PVT neuron activity affects the induction times of both sevoflurane and desflurane anesthesia. Differences in electroencephalographic (EEG) results during the induction and recovery phases suggest that the regulatory mechanisms of PVT glutamatergic neurons may vary during sevoflurane-induced unconsciousness and recovery. Additionally, during mild sevoflurane anesthesia, anesthesia induction is delayed by the activation of glutamatergic neurons along the pathway from the PVT to the bed nucleus of the stria terminalis (BNST) [69]. Furthermore, inhibiting the PVT or its pathways during sevoflurane anaesthesia may exacerbate hyperpolarization and accelerate cortical synchronization, thereby promoting loss of consciousness. Optogenetic activation of GABAergic neurons in the zona incerta (ZI) enhances the anesthetic effects of sevoflurane; however, there was no significant effect on anesthesia maintenance [70]. During sevoflurane anaesthesia, there is a specific disruption of functional connectivity between the thalamus and the frontal cortex (Fig. **2**) [13, 15].

In summary, the cortex plays a significant role during the administration of volatile anaesthetics. Interestingly, studies have shown that during induction with volatile anaesthetics, injury to glutamatergic neurons in the anterior piriform cortex (APC) shortens the induction time [71]. In contrast, no such effect is observed with intravenous anaesthetics. This further supports the hypothesis that volatile anaesthetics may exert their effects *via* the olfactory pathway. During anaesthetic induction, each nucleus and neurotransmitter is of critical importance. The PVT is a key nucleus in controlling arousal, and existing experimental data suggest that it also plays a role during induction. The hypothesis that the PVT may exert a controlling effect during the induction process of volatile anaesthetics cannot be dismissed. Additionally, the relationship between the analgesic effects of volatile anaesthetics and the spinal cord [72] should not be overlooked.

### 2.2 Intravenous Anaesthetics

Intravenous anaesthetics are drugs administered *via* intravenous injection or infusion. GABAergic drugs, [73] such as barbiturates and chloral hydrate, are used to induce loss of consciousness. Currently, the main intravenous anaesthetics used clinically include propofol, ketamine, dexmedetomidine, and sodium barbiturate. The specific mechanisms of these drugs in anaesthesia induction are not yet fully understood.

#### 2.2.1 Propofol

Propofol, a widely used intravenous general anaesthetic, induces sleep by inhibiting the levels of c-Fos in multiple nuclei of the arousal system, including the TMN, PeF, LH, and vPAG, while activating sleep-promoting clusters in the VLPO [74]. Although studies have indicated that propofol affects the LC-NE system, the PnO, and the mesopontine tegmentum anaesthesia area (MPTA, found in the upper region of the brainstem), and significantly impacts cholinergic and dopaminergic neurons in regions such as the PrL, BF, and NAC, the specific neural circuit mechanisms involved in its induction are still not well understood. For example, exposure to propofol suppresses activity in the LC [21, 75]. Recent research using zebrafish larvae as an anaesthesia model has demonstrated that propofol-induced anaesthesia is severely affected by LC neuron lesions and norepinephrine release depletion, highlighting the critical role of the LC-NE system in propofol anaesthesia [76]. Additionally, microinjection of GABA synthesis inhibitors (3-mercaptopropionic acid) or GABA uptake inhibitors (nipecotic acid) into the head-side PnO only promotes propofol anaesthesia induction, but it does not affect the wake-up time [77]. Bilateral VTA lesions substantially increased recovery time from propofol anaesthesia, but had no impact on induction time, and neither induction nor awakening was notably affected by ketamine [28]. The MPTA is considered a crucial site for the induction and maintenance of anesthesia [78, 79] and is also a primary site of action for GABAergic drugs in general anesthesia induction. Damage to the MPTA region decreases sensitivity to GABAergic anaesthetics [80].

During propofol anaesthesia, loss of function in BF cholinergic neurons [30, 81, 82] shortens the induction time and enhances the anaesthetic effect, whereas activation of dopaminergic neurons can reverse the anaesthetic sedation. Additionally, the lack of effect of cholinergic lesions on halothane sensitivity also indicates that the stimulation of cholinergic neurons is involved in mediating propofol and pentobarbital anaesthesia, rather than solely promoting hypnosis through non-specific mechanisms [21]. However, injecting D_1_R agonists into the basal nucleus (NB) of the BF does not affect anaesthesia induction but accelerates recovery from propofol anaesthesia [4] Studies using ibuprofen (NSAID) injection into the BF have found that BF neuron activity is suppressed during propofol anaesthesia induction [83]. In the case of propofol anaesthesia, D1R-mediated inhibition of NAc neurons occurs during induction. D_1_R affects the activity of NAc MSNs, which contain abundant GABAergic medium spiny neurons, through presynaptic mechanisms, playing an important role in propofol-induced unconsciousness [84]. Moreover, a reduced sensitivity to propofol, along with a prolonged induction time and facilitated emergence from anaesthesia, has been observed following chemical activation of GABAergic neurons in the NAc [33], highlighting their critical role during propofol induction.

The DA neurons within the vPAG participate in the induction processes of propofol anaesthesia [85]. Damage to these neurons produces the opposite effect, leading to a shortened induction time and an extended recovery time compared to normal function. Propofol activates the VLPO through metabolism, a crucial mediator of propofol-induced unconsciousness [36, 37, 86]. Studies showed that administering different doses of propofol to VLPO-lesioned and sham-lesioned mice demonstrates that GABAergic neurons in the VLPO mediate propofol-induced general anaesthesia [87]. The regulation of GABAergic transmission to VLPO neurons mediated by M1 receptors may play a role in the induction process of isoflurane [88]. Therefore, it can be inferred that propofol induces anaesthesia by activating GABAergic neurons in the VLPO, which indirectly inhibits dopaminergic neurons in the vPAG [85]. However, it has been confirmed that DRN 5-hydroxytryptamine neurons are not significantly affected during the induction of propofol general anaesthesia [89]. Moreover, VLPO has been shown to be essential for the suppression of LC activity induced by propofol [90]. Consistent with previous findings, the VLPO contributes to the loss of consciousness by inhibiting arousal-promoting nuclei.

Research has confirmed that propofol anaesthesia can inhibit the activity of hypothalamic orexin neurons [91]. Activation of these neurons promotes awakening from propofol anaesthesia, but their relationship to the induction process remains unclear. Additionally, the administration of propofol results in membrane hyperpolarization in GABAergic neurons of the LH, [92], suggesting that LHA GABAergic neurons play a role in propofol-induced anaesthesia. Further studies have shown that the induction of propofol anaesthesia is facilitated by the inhibition of glutamatergic neurons in the LH [93]. Specifically, the activation of glutamatergic neurons has been found to prolong the induction time, whereas the role of GABAergic neurons in the induction of anesthesia remains unclear. Studies on the effects of propofol on voltage-gated Ca^2+^ currents have shown that propofol inhibits PVH neurons by enhancing GABA-A receptor-mediated currents [94]. The LHb is involved in sleep regulation and the effects of propofol anesthesia [43, 95]. One study found that the activity of excitatory neurons in the LHb is necessary for the sedative effects induced by propofol, and damage to LHb glutamatergic neurons weakens the anaesthetic induction effect of propofol. Conversely, anaesthesia is accelerated by the selective activation of the LHb. Research establishing a novel thalamocortical model suggests that isoflurane may induce hypnosis at least partly through its action on the thalamus. Furthermore, the activation of GABA-A receptors induced by isoflurane facilitates thalamic engagement and promotes cortical synchronisation. Descriptions of the righting reflex indicate that the periventricular thalamus plays a role in controlling consciousness transitions during propofol anaesthesia [96]. Notably, a recent study [97]. reported that activation of glutamatergic neurons in the paraventricular thalamus during optogenetic experiments resulted in a prolongation of the anaesthetic induction time. However, the effect was not statistically significant. Extracellular recording studies have shown that neuronal activity in the ventral posteromedial nucleus (VPN) is suppressed by propofol, reducing signal transmission to the primary somatosensory cortex barrel field (S1BF) [98]. Multisite intracranial local field potential (LFP) recordings and fast-time domain analysis indicate the central medial thalamus (CMT) as a key hub for initiating anaesthesia-induced unconsciousness under propofol anaesthesia [99]. Additionally, microinjection of norepinephrine into the CMT can accelerate recovery from propofol anaesthesia but does not affect induction [100]. The role of CMT in propofol anaesthesia induction remains unconfirmed. Studies have indicated that activating GABAergic neurons in the ZI may promote the induction effects of propofol anaesthesia without significantly affecting maintenance [70]. Moreover, stimulation of GABAergic neurons in the anterior thalamic reticular nucleus (aTRN) has been shown to promote induction and delay recovery from propofol anaesthesia [45]. Interestingly, the TRN sends significant GABAergic projections to other thalamic nuclei, which serve to synchronize the different relay nuclei and thereby facilitate synchronization across large cortical areas [101]. It mediates information exchange between the thalamus and cortex during the induction process, playing a crucial role as a bridge in the thalamocortical network.

**Fig. (3):**
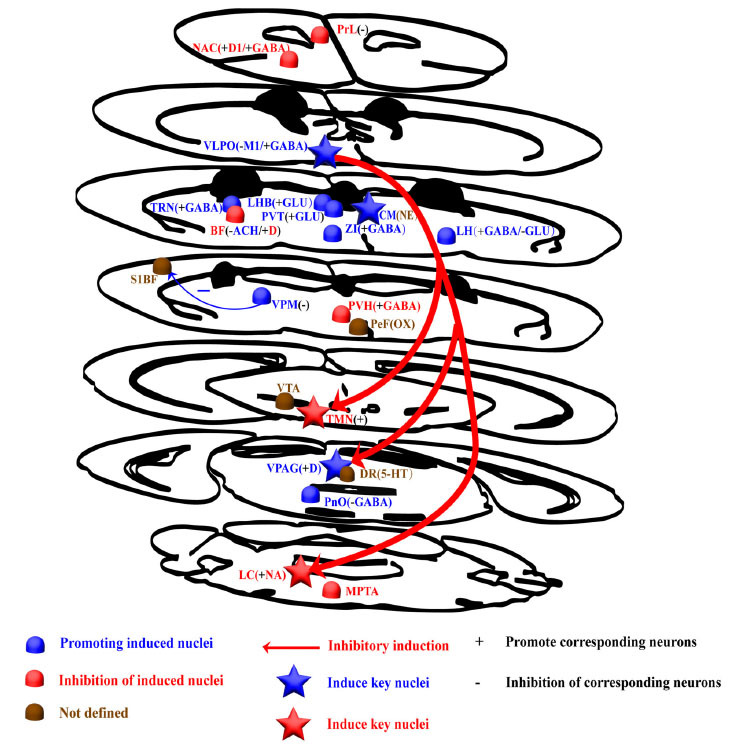
Neuronal connections between cortical and subcortical nuclei during propofol-induced general anaesthesia. Within the CM, brown NE indicates that NE does not affect the induction process under propofol anaesthesia.

Previous research [86] has shown that propofol can disrupt the connectivity between the thalamus and the lateral frontal-parietal network. The unconsciousness induced by propofol may be related to the collapse of temporal lobe structures, which alters connections within and between networks, preventing communication between lower-order sensory regions and higher-order frontal-parietal cortices. Positron emission tomography has indicated that the hypnotic effects of propofol may be associated with decreased activity in the thalamus and precuneus [102], suggesting that these regions are important for the loss of consciousness caused by propofol. Measurement of deoxyhemoglobin concentrations in the prefrontal cortex (PFC) reveals that during the induction of propofol anesthesia, the bispectral index (BIS) value decreases, and PFC activity generally declines [103], indicating a connection between PFC activity and consciousness. Blocking GABA-A receptors in the medial PFC can alter the timing of LORR and RORR, leading to delayed induction time and a shortened recovery time from propofol anaesthesia [104], indicating that different neurotransmitters within the same cortex have varying effects during anaesthesia induction. Notably, the endocannabinoid system [105, 106] contributes to the sedative properties of propofol, enhancing the induction process (Fig. **3**).

In summary, the mechanisms underlying propofol general anaesthesia during induction are closely related to the thalamocortical network, with interactions among various nuclei and their neurotransmitters collectively shaping the anaesthesia induction process. However, anaesthesia is not solely dependent on brain nuclei; it also involves the peripheral nervous system. Previous studies have shown that propofol has a direct inhibitory effect on spinal cord neurons, acting on GABA and glycine receptors in acutely isolated spinal dorsal horn neurons [107].

#### 2.2.2 Ketamine

**Fig. (4):**
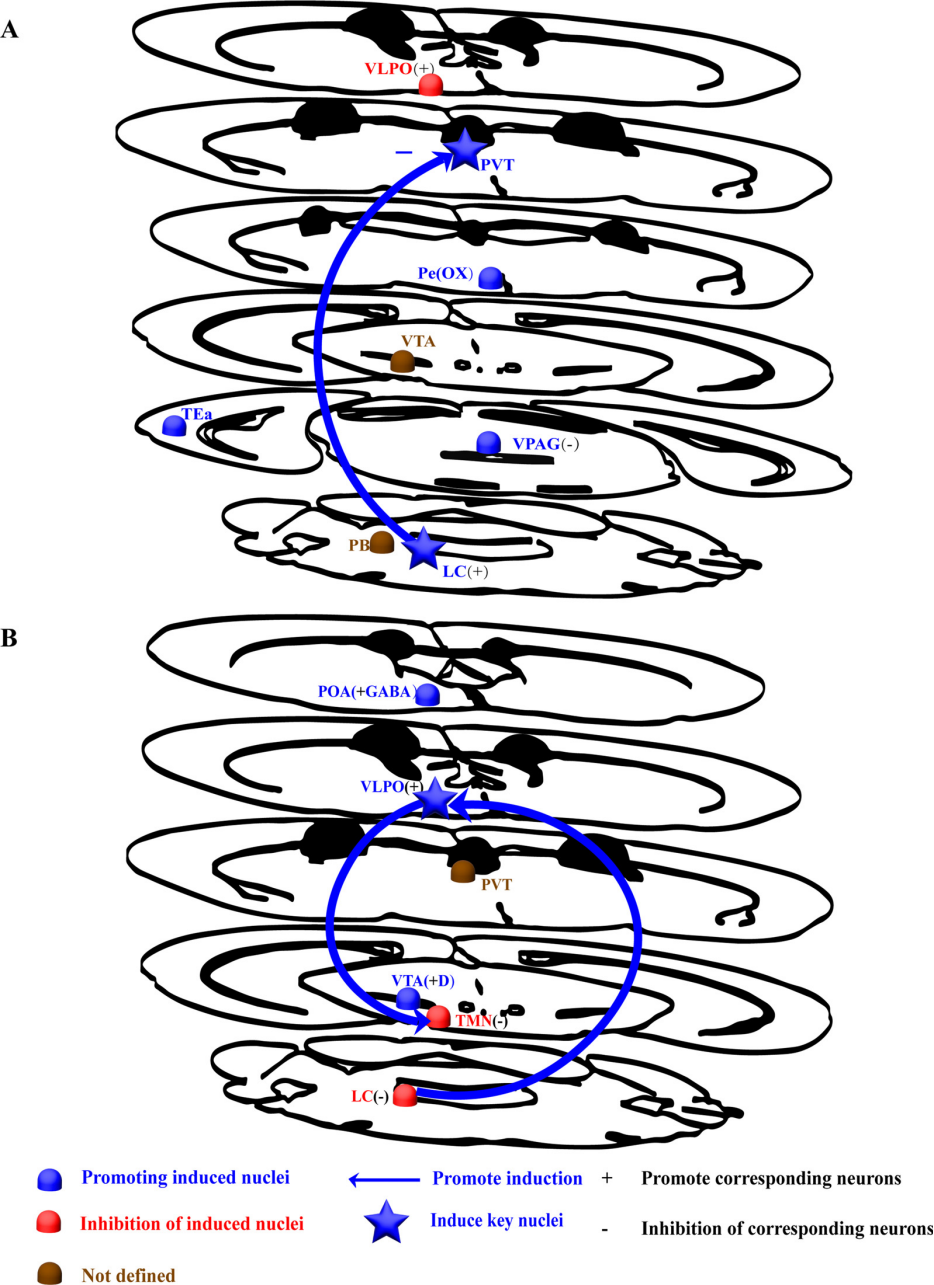
Neuronal connections between cortical and subcortical nuclei during general anaesthesia induced by other anaesthetics. (**A**) Ketamine-induced general anaesthesia. (**B**) Dexmedetomidine-induced general anaesthesia.

Ketamine is a unique anaesthetic that produces loss of consciousness, analgesia, and amnesia. It is used for both intravenous anaesthesia and as an antidepressant. A notable feature of ketamine is its ability to separate hypnotic and analgesic effects. As an anaesthetic, ketamine works by inhibiting NMDA receptor-mediated glutamate input, altering the excitability of the thalamus, cortex, and limbic system, eventually leading to loss of consciousness (Fig. **4A**).

Ketamine preferentially inhibits excitatory inputs to GABAergic interneurons mediated by NMDA receptors, leading to dysregulated excitatory activity, particularly in the cortex, hippocampus, and limbic system, which ultimately results in loss of consciousness [1]. Ketamine has been shown to activate various brain regions, particularly the cortex, with the temporal association area (TEa) serving as a central node in its functional network, indicating that ketamine's mechanism of action is top-down. Studies have demonstrated that its hypnotic effect is mediated, at least in part, through activation of the LC [21, 73, 108]. The anaesthetic effects are linked to increased activity in the LC, which inhibits the anaesthetic action of thiopental while enhancing the effects of ketamine. Additionally, ketamine's anaesthetic action may be related to the HCN1 pacemaker channel, with HCN1 subunits considered molecular substrates for its hypnotic effects [109]. Despite its hypnotic effects, ketamine seems to inhibit activation of the VLPO [73], possibly due to its primary action on NMDA receptors. It has been shown that the full anaesthetic effects induced by ketamine significantly reduce the firing rate of GABA neurons within the VTA, [27], although damage to the VTA’s DA neurons has no effect on ketamine's anaesthetic effects [28]. Studies in Sprague-Dawley rats suggest that orexinergic neurons may be significant targets for ketamine, with orexin A (OXA) neurons reducing the duration of ketamine-induced anesthesia [110]. Glutamate receptors are important targets for ketamine's induction of consciousness loss and analgesia during surgery [111]. Ketamine may lower cortical arousal levels by straightforwardly acting on the thalamus and inhibiting glutamatergic connections from the parabrachial nucleus to the thalamus, thus reducing arousal by inhibiting excitatory interneurons. The PVT [46] and the dorsal lateral thalamic group (LAT) [112] show enhanced activity under the influence of ketamine [108]. One study demonstrated that ketamine weakens glutamatergic neurotransmission by inhibiting postsynaptic NMDA receptors located in the primary somatosensory cortex and the VPM [113, 114], suggesting that the primary site of ketamine's action within the central nervous system may be the thalamocortical system. During general anaesthesia, ketamine achieves loss of consciousness by disrupting the connections between these regions. Research has shown that ketamine primarily induces cortical activation, with a reduction in the latency of anaesthesia occurring only in animals with damage to the VPAG [73].

The method by which ketamine induces unconsciousness has been successfully compared with the synaptic actions of other anaesthetics, including propofol and sevoflurane [37]. Compared to propofol, ketamine's inhibitory effects on nicotinic receptors may not be directly related to its sedative effects, but rather may play a role in its analgesic properties [115]. Notably, ketamine's analgesic effects on the spinal cord may be achieved through widespread suppression of neuronal activity in the dorsal horn, and the loss of consciousness it induces is closely related to mechanisms involving dorsal horn neurons in the spinal cord.

#### 2.2.3 Esketamine

Esketamine, the S-enantiomer of ketamine, exhibits stronger NMDA receptor antagonism and is more effective in treating depression. Observations indicate that the administration of esketamine elevates c-Fos levels in the PVT region of mice, suggesting the activation of PVT glutamatergic neurons. Such activation has been found to reverse sevoflurane-induced anaesthesia and accelerate recovery [116, 117]. These neurons are also considered important structures in the transition to an anaesthetic state. As a novel anaesthetic, esketamine has limited clinical application. Research can be conducted on its effects on the cortical and subcortical nuclei, further elucidating its anaesthetic mechanisms.

#### 2.2.4 Dexmedetomidine

Dexmedetomidine is a selective α2-adrenergic receptor agonist primarily used for sedation and anaesthesia management, with effects associated with the VLPO and VTA. Studies have confirmed that dexmedetomidine induces loss of consciousness through interaction with neurons in the POA [118]. Selective removal of the vesicular GABA transporter gene (VGAT) in the POA slows the rapid transition to a sedated state following dexmedetomidine administration; however, mice remained sedated for 30 minutes post-injection. This suggests that GABAergic neurons in the POA are crucial for the rapid induction but not necessary for its maintenance [119]. One study shows that dexmedetomidine lowers the arousal threshold during sedation by activating dopamine neurons in the VTA, [120], which may relate to its unique sedative characteristics that allow for easy arousal of patients.

Dexmedetomidine induces general anaesthesia by inhibiting the LC projections to other brain regions, decreasing consciousness levels and inducing EEG changes resembling to NREM sleep—specifically, inactivating the LC and the TMN, while activating the VLPO [121]. Dexmedetomidine suppresses LC activity and reduces LC projections to the VLPO, [21] consistent with the effects of other anaesthetics. Additionally, TMN inhibition reduces histamine release in cortical and subcortical projection areas. This is also linked to the thalamocortical network, suggesting that the LC-VLPO-TMN pathway may be one of the mechanisms underlying dexmedetomidine-induced loss of consciousness. Additionally, dexmedetomidine affects the projections from the LC to the thalamic intralaminar nuclei, the BF, and the cortex, thereby disrupting thalamocortical functional connectivity —a primary mechanism for inducing unconsciousness [122]. However, its effects on specific thalamic nuclei and the spinal cord remain unclear (Fig. **4B**).

#### 2.2.5 Etomidate

As an intravenous anesthetic, etomidate is primarily used for induction of anesthesia or rapid anesthesia, enhancing GABA-A receptor function. Research indicates that the LC-NE system may play different roles at various stages of etomidate anaesthesia [76] with this system being in an inhibitory state during the induction phase. Furthermore, the observation of EEG signals during general anaesthesia induction in clinical patients [123] indirectly reflects the involvement of the cerebral cortex and even brain nuclei in the induction process, providing new directions for basic experimental research.

#### 2.2.6 Benzodiazepine Drugs

The benzodiazepine anaesthetic, midazolam, induces anaesthesia by selectively acting on GABA-A receptors [86]. Furthermore, a study investigating the effect of midazolam on recovery time from general anesthesia in mice with GABA receptor knockouts in the VLPO region [124] indirectly suggests that it may influence the duration of anesthetic induction. Localised lesions in the CMT significantly shortened the time to loss of consciousness induced by diazepam and blocked cortical information integration by inhibiting thalamic activity [125], thereby affecting signal transmission within the thalamocortical loop and between subcortical nuclei.

#### 2.2.7 Barbiturates Drugs

Clinically, barbiturate drugs include pentobarbital and thiopental. Previous studies suggest that the LC plays a key role in thiopental-induced anaesthesia [21]. General anaesthesia induced by pentobarbital injection significantly reduces the density of FOS-immunoreactive nuclei in the MPTA [126]. Moreover, accurately targeting 10 mg of pentobarbital in this area may suffice to induce anaesthesia, leading to analgesia, muscle relaxation, and loss of consciousness [77]. This indicates its significant impact on the induction of anaesthesia in this region. OXA significantly shortens the anaesthesia time for thiopental, pentobarbital, and phenobarbital [110]. While existing studies have revealed some mechanisms of barbiturate anaesthesia, further research could focus on the mechanisms of general anaesthesia induction involving other subcortical nuclei.

#### 2.2.8 Opioids

During anaesthesia, opioids are primarily used for analgesia, with the potential loss of consciousness due to overdose not being considered at this stage. It is worth noting that opioids can achieve analgesia by inhibiting mechanically gated channels in the spinal dorsal horn [127]. This provides new insights into peripheral mechanical pathways for studying the mechanisms of anaesthetic induction.

## 3 APPLICATION OF FUNCTIONAL BRAIN IMAGING TECHNIQUES IN THE STUDY OF NEURAL CIRCUITS DURING GENERAL ANAESTHESIA

General anaesthesia finely regulates the neural circuits in the cortical and subcortical regions of the brain to control both consciousness and physiological states, while maintaining the stability of essential vital signs. Anaesthetics act on brainstem circuits to regulate respiration and cardiovascular function, ensuring the normality of vital signs [2]. The development of neuroscientific techniques, such as optogenetics and chemogenetics, has facilitated research into the neural circuit mechanisms of general anaesthesia [19]. Despite recent advancements at the molecular level, it has been concluded that the common endpoint of different anaesthetics is the disruption of cortical information integration [128]. At the brain level, how anaesthetics regulate the connections between different brain regions, particularly in coordinating the dynamic interactions between these regions, requires further in-depth investigation. The understanding of how anaesthetics coordinate across various brain regions, particularly the impact on neural circuits when combining different anaesthetics (such as the combination of ketamine and isoflurane [108]), remains a complex and unresolved mystery.

Functional brain imaging techniques, such as EEG, positron emission tomography (PET), functional magnetic resonance imaging (fMRI), and intraoperative neurophysiological monitoring (IONM), provide essential tools for further exploring these mechanisms. The EEG [19] measures the overall activity of the cerebral cortex, reflecting the activity of local neurons and the connectivity between brain regions. During general anaesthesia, the EEG shows characteristic waveform changes, particularly in the frontal lobe region. It has been demonstrated that [129] in the awake state, the EEG shows high-frequency, low-amplitude, and asynchronous activity. At the same time, during anaesthesia-induced loss of consciousness, it displays low-frequency, high-amplitude, and synchronous activity. For example, during ketamine-induced general anaesthesia, the specific slow δ waves and fast γ wave activity observed in the EEG, along with the alternating δ and γ wave patterns, reflect changes in brainstem modulation of thalamic and cortical output signals. Additionally, combined EEG and PET studies have found that sevoflurane induces significant changes in cerebral blood flow and metabolic activity in regions such as the frontal lobe, parietal lobe, and thalamus [130]. These disturbances may play a central role in the transition between anaesthetised and awake states. The fMRI provides us with imaging techniques of high spatial resolution, allowing us to avoid the confounding effects of tracers and observe in real-time the activity changes across the entire brain, including both cortical and subcortical regions. During the induction of general anaesthesia, the activity of higher cortical or associated cortical areas is initially suppressed, followed by a reduction in the response of primary cortical regions [131]. Current research suggests that anaesthetics can alter the functional connectivity between the cortex and subcortical nuclei during the resting state. During the loss of consciousness induced by sevoflurane anaesthesia, fMRI technology revealed changes in the connectivity patterns between key brain regions. For example, sevoflurane may alter the connectivity of higher-order cortical regions, particularly the default mode network, which are crucial for cognitive function during wakefulness [128, 132]. During the loss of consciousness induced by propofol anaesthesia, the overall information exchange within the functional brain network is enhanced, and changes also occurred in the connectivity between the temporal and frontal cortical regions [133]. Furthermore, resting-state fMRI has further revealed the impact of anaesthetics on thalamic and subcortical network functional connectivity, particularly the lateralisation and severe limitation of subcortical functional connectivity [8]. Huang and colleagues [134] demonstrated that, during propofol-induced anaesthesia, unconsciousness can be triggered by disrupting the cross-modal connectivity between thalamic core and matrix cells. This pioneering work bridges the gap between cellular-level mechanisms and system-level functional connectivity. Concurrently, Chen and colleagues [135] employed multimodal imaging techniques, revealing that suppression of the hippocampus-thalamus-mPFC pathway may be a key mechanism underlying loss of consciousness, further supporting the thalamocortical loop theory in the context of general anesthesia-induced unconsciousness. In addition, IONM [136], through real-time electrophysiological recordings of specific neural pathways-such as somatosensory evoked potentials (SSEPs) and motor evoked potentials (MEPs)-provides a millisecond-resolution, dynamic electrophysiological complement to the functional connectivity changes observed *via* fMRI.

By combining functional brain imaging techniques, [137] researchers can more comprehensively explore how anaesthetics induce loss of consciousness by disrupting the interactions between cortical, thalamic, brainstem, and other brain regions. In particular, fMRI technology can quantify how anaesthetic agents alter the functional connectivity and information transfer between different brain regions, revealing the neural circuit mechanisms underlying the loss of consciousness. For example, by using the integrated-segregated difference (ISD) index in fMRI, researchers can accurately identify the transition from wakefulness to unresponsive states [138], thereby exploring the mechanisms of anaesthetic agents within the brain network. During the process of unconsciousness induced by propofol, functional brain imaging techniques have shown a significant reduction in the efficiency of rich-club nodes in the insula, precentral gyrus, posterior cingulate cortex, and cerebellum [139]. This change indirectly reflects variations in the depth of anaesthesia.

In conclusion, combining neural circuit mechanisms with brain functional imaging techniques allows for a deeper understanding of how anaesthetics affect various brain regions and neural circuits. This provides us with a dynamic perspective, not only helping us understand how anaesthetic agents induce loss of consciousness, but also offering new theoretical insights for the optimisation of anaesthetic depth control and the combined use of drugs.

## 4 CLINICAL SIGNIFICANCE

Regulating anaesthetic depth has always been a core issue in clinical anaesthesia. With a deeper understanding of the neural circuit underlying general anesthesia, we may be able to more precisely control the use of general anesthetics, ensuring that the depth of anesthesia is sufficient to achieve loss of consciousness and meet the requirements of surgery, while also reducing anesthesia-related risks and adverse effects. In this process, the Higher-Order Theory (HOT) helps us better understand the critical point of consciousness loss and its relationship with anaesthetic depth. The HOT posits that consciousness arises from an individual's perception of their mental representations in a particular state, which requires complex activity in the higher cortical areas of the brain [20]. Research focusing on both the cortical and subcortical nuclei is a crucial pathway to understanding the mechanisms of general anaesthesia. By studying the mechanisms of general anaesthesia, particularly the effects of general anaesthetics on neural circuits, we not only aid in the precise regulation of individual anaesthetic depth but also deepens our understanding of the mechanisms of consciousness.

## 5 LIMITATIONS

This review synthesizes the neural circuit mechanisms underlying general anesthesia induction; however, several limitations must be acknowledged. First, mechanistic insights predominantly rely on rodent models, in which neuroanatomical differences (such as thalamic nuclear differentiation and cortical laminar organization) may overemphasize the role of specific nuclei (such as the PVT) in humans. Although human neuroimaging partially bridges this gap, the absence of cross-species validation platforms hinders clinical translation. Second, the overreliance on LORR fails to disentangle multidimensional anaesthetic endpoints (unconsciousness, analgesia, amnesia), potentially conflating cortical-thalamic suppression with spinal motor pathway inhibition. Furthermore, while most studies focus on single agents (such as propofol and sevoflurane), the synergistic mechanisms of clinically prevalent polypharmacy regimens (such as propofol-opioid combinations) remain poorly understood, particularly the dynamic interplay between GABAergic and opioid receptor signaling in thalamocortical circuits. Lastly, intraoperative monitoring techniques (such as EEG and fMRI) lack sufficient spatiotemporal resolution to pinpoint active nuclei (such as the LC) in real-time.

## 6 FUTURE RESEARCH PRIORITIES

Different types of anaesthetic agents affect neural circuits through fundamentally distinct mechanisms. (Table **1**) GABAergic agents [140]—such as isoflurane, sevoflurane, propofol, etomidate, benzodiazepines, and barbiturates—enhance GABA-A receptor-mediated chloride influx, thereby strengthening inhibitory synaptic transmission. These agents preferentially suppress excitatory inputs within cortico-thalamic circuits, induce globally synchronised low-frequency oscillations (notably delta waves), and disrupt information integration in higher-order cortical networks, such as the fronto-parietal network. For instance, propofol selectively enhances GABA-A receptor function [141] while isoflurane and sevoflurane, in addition to acting on the GABAergic system, can inhibit glutamate release, thereby indirectly reducing cortical excitability. Luo *et al*. proposed that volatile anaesthetics-including isoflurane and sevoflurane intravenous agents-namely propofol and etomidate-may suppress the “consciousness switch” function of glutamatergic neurons [142]. In contrast, NMDA receptor antagonists (such as ketamine and esketamine) block NMDA receptor-mediated calcium influx, thereby inhibiting cortical GABAergic interneurons and relieving their inhibitory control over glutamatergic neurons. This results in cortical hyperexcitability and decoupling between the thalamus and cortex. The core mechanistic distinction lies in the fact that GABAergic anaesthetics reduce overall neural excitability by enhancing inhibitory tone. In contrast, NMDA antagonists disrupt the excitation-inhibition balance, leading to network-level destabilisation. Additionally, propofol may enhance its analgesic effect by inhibiting GABAergic pathways in the dorsal horn of the spinal cord. In contrast, ketamine’s spinal actions are primarily mediated through modulation of nociceptive signalling *via* NMDA receptors.

Although comprehensive studies covering all nuclei and anaesthetics are lacking, some common themes emerge. For instance, pallido-cortical connectivity is essential for the loss of consciousness during anesthesia induction [143]. The periorbital area (PZ) [34] promotes loss of consciousness by inhibiting additional arousal systems. Except for ketamine, the LC is typically inhibited during general anaesthesia induction, while the VLPO facilitates the unconscious state induced by general anaesthesia. The VTA-DA neurons are not essential for anaesthesia induction, whereas the inhibition of cholinergic neurons in the BF is related to loss of consciousness. DA neurons in the vPAG can influence anaesthesia effects. The PVT is crucial for consciousness transition, and inhibiting the PVT-BNST pathway shortens induction time [53]. Activation of GABA neurons in the TRN can facilitate the induction of anesthesia.

Some studies have proposed that the cortico-thalamo-cortical circuit, along with the prefrontal cortex, may constitute the key neural mechanisms underlying loss of consciousness during general anaesthesia [129]. If anaesthesia induces loss of consciousness by suppressing cortical information integration, then the thalamus may, in fact, represent the primary target of this suppression [99]. The thalamus is composed of more than 50 neuronal nuclei and subnuclei, which can generally be classified into two categories: relay stations that receive peripheral sensory inputs and multimodal integration areas that receive cortical inputs. Additionally, the thalamus plays a crucial role in relaying brainstem arousal signals and regulating communication with the cerebral cortex. It is critical for arousal, sensory processing, and cortical information integration, potentially acting as a switch for consciousness.

Research indicates that the PVT plays an important role in controlling arousal, with sustained activation of PVTGlu neurons accelerating the onset of unconsciousness induced by anaesthesia [144]. Although many studies suggest that arousal and loss of consciousness are opposing processes, and that sleep-wake nuclei may be involved in both anesthesia-induced loss of consciousness and recovery, experiments have shown that anesthesia induction and awakening are separate processes with distinct neural circuits [4, 77]. Whether these processes are completely distinct is not yet fully proven. The PVT may also play a crucial role in controlling loss of consciousness, with anaesthetics potentially functioning by blocking the transmission of information from the paraventricular thalamus to other nuclei.

**Fig. (5):**
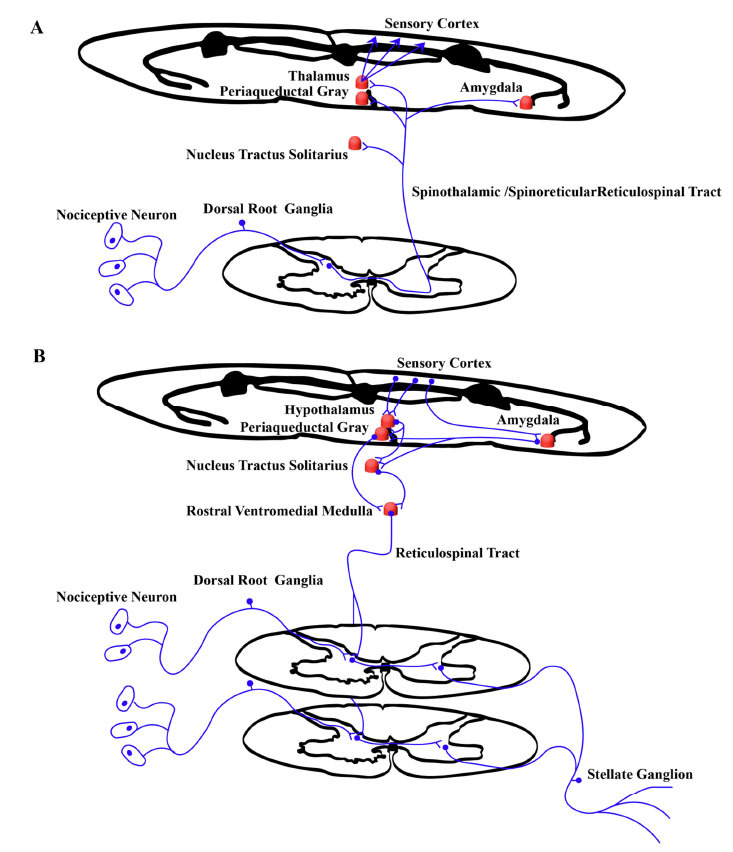
In a normal state, the ascending and descending pain pathways operate as follows: (**A**) The ascending nociceptive pathway. (**B**) The descending nociceptive pain.

Noxious signals are converted and transmitted to the dorsal root ganglion (DRG), [145-147] subsequently ascending through the spinal dorsal horn *via* the spinothalamic tract to the thalamus, and finally relaying to the somatosensory cortex of the brain (Fig. **5A**). The descending sensory pathway begins in the cortical areas, with signals passing through the hypothalamus, amygdala, LC, vPAG, and the reticulospinal tract, projecting to the spinal cord to modulate the processing of pain signals [148]. The stellate ganglion, a part of the sympathetic nervous system, is involved in the sensation and control of visceral organs (Fig. **5B**). Current research into the mechanisms of general anesthetic induction predominantly centers on the central nervous system, specifically the cortical and subcortical nuclei, as well as the spinal cord [149]. Future research should prioritize investigating central-peripheral neural interactions, particularly how spinal-thalamic pathways mediate the dynamic relationships between peripheral sensory signaling and cortical integration of consciousness. By integrating single-cell sequencing with multimodal neuroimaging techniques, it will be possible to delineate subtype-specific neuronal connectivity from the spinal cord and brainstem to the cortex, thereby elucidating principles of peripheral-central signal transduction. Concurrently, computational modelling approaches that simulate drug-induced perturbations across hierarchical circuits (such as spinal microcircuits and thalamocortical networks) could transcend conventional brain-centric paradigms, advancing a unified framework for the systemic regulation of anaesthesia.

## CONCLUSION

The induction of general anaesthesia involves multiple neural circuits spanning both the central and peripheral nervous systems. GABAergic agents and NMDA receptor antagonists induce loss of consciousness through distinct mechanisms-either by enhancing inhibitory signalling or disrupting the excitation–inhibition balance. Key central structures such as the thalamus, subthalamic nucleus, and cortico-thalamic circuits play a pivotal role, with the spinal cord and peripheral sensory pathways equally critical. Increasing evidence suggests that anaesthetic induction and emergence are not merely opposing phases of a single process, but instead distinct phenomena governed by separate neural dynamics. Future research should integrate advanced neurotechnological tools and computational modeling to elucidate central–peripheral interactions, establishing a systems-level framework for anaesthetic induction and guiding the development of more reliable and precise anaesthetic strategies.

## Figures and Tables

**Table 1:** Activation and inhibition effects by anesthetic agents. regulation of neuronal nuclei and pathways and impact on anesthetic induction time.

**-**	**Activation of Neuronal Nuclei Through Neurotransmitter** **Signalling Shortens Anaesthetic Induction Time**	**Inhibition of Neuronal Nuclei** **Through Neurotransmitter Signalling Shortens Anaesthetic Induction Time**	**Activation of Neural Pathways Shortens Anaesthetic Induction Time**	**Inhibition of Neural Pathways Shortens Anaesthetic Induction Time**
ISO (GABA)	MnPO, VLPO, POA, LHB-GLU, PVT-GLU, TRN-GABA, BF-GABA/AST, LH, VTA-GABA, VPAG-GABA, RMTG-GABA	APC-GLU, NAC-GABA, PVH-GLU, PnO-ACH/GABA, LC-NA	LHB→RMTg, VTA→LH	-
SEVO (GABA)	SI-GLU, ZI-GABA, VTA-DA, TMN-GABA, RMTG-GABA	APC-GLU, NAC-DA, MPA-ERa, PVT-GLU, DMH-GABA, PPTg-GLU, PB-GLU	NAC→VP	NAC→VP, PVT→BNST, TMN→MS →PrL
PROP (GABA)	PrL, VLPO-GABA, LHB-GLU, ZI-GABA, TRN-GABA, LH-GABA, VPAG-D	VLPO-M1, BF-ACH/D, LH-GLU, CM, PVH-GABA, VPM, PVT-GLU PnO-GABA, LC-NA, MPTA	-	→TMN VLPO→VPAG →LC
KET (NMDA)	PVT-GLU, PeF-OX, TEa, LC	VLPO, VPAG	LC→PVT	-
DEX	POA-GABA, VLPO, VTA-D, TMN, LC	-	LC→VLPO→TMN	-
ETO (GABA)	-	LC-NE	-	-
BZDs (GABA)	-	CMT, LC-NE	-	-
BARBs (GABA)	-	MPTA	-	-

## LIST OF ABBREVIATIONS

BF: Basal Forebrain
BIS: Bispectral Index
CMT: Central Medial Thalamus
DRN: Dorsal Raphe Nucleus
IONM: Intraoperative Neurophysiological Monitoring
LC: Locus Coeruleus
LH: Lateral Hypothalamus
NAC: Nucleus Accumbens
PET: Positron Emission Tomography
PFC: Prefrontal Cortex
PVT: Paraventricular Thalamus
vPAG: Ventral Periaqueductal Gray
VPN: Ventral Posteromedial Nucleus
VTA: Ventral Tegmental Area
